# Multidrug-resistant tuberculous orchiepididymitis: a brief case report

**DOI:** 10.1590/S1678-9946202365061

**Published:** 2023-12-04

**Authors:** César Augusto Tomaz de Souza, Jussemara Souza da Silva, Ademir Silva Correia, Denise Silva Rodrigues

**Affiliations:** 1 Instituto Clemente Ferreira, Departamento de Infectologia, São Paulo, São Paulo, Brazil; 2 Instituto de Infectologia Emílio Ribas, Departamento de Infectologia, São Paulo, São Paulo, Brazil; 3 Instituto de Infectologia Emílio Ribas, Divisão de Apoio ao Diagnóstico e Terapêutica, Seção de Radiologia, São Paulo, São Paulo, Brazil

**Keywords:** Testicular tuberculosis, Multidrug-resistant tuberculosis, Orchiepididymitis, Extrapulmonary tuberculosis

## Abstract

Tuberculosis (TB) is one of the leading causes of death by infectious diseases worldwide. Multidrug-resistant tuberculosis is a growing problem, especially in countries with high TB prevalence. Although the lungs are the organs most frequently affected by this disease, *Mycobacterium tuberculosis* can harm any organ, including the urogenital tract, causing extrapulmonary tuberculosis, which leads to a challenging diagnosis and consequent treatment delays. In this article, we present a case of orchiepididymitis caused by multidrug-resistant TB (MDR-TB) with a significantly delayed diagnosis, the proposed treatment according to the resistance profile, and the clinical outcomes.

## INTRODUCTION

Tuberculosis (TB) is one of the most common causes of death by infectious diseases worldwide, and multidrug-resistant tuberculosis (MDR-TB) is a growing problem in developing countries, including Brazil. Extrapulmonary tuberculosis (EPTB) can affect many body systems, including the urogenital tract, in which the kidneys are the most affected organs.

Genitourinary TB can affect the kidney, ureter, bladder, epididymitis, prostate, and testes. TB epididymitis can originate in the kidneys or be hematogenously disseminated from the lungs. The variety of symptoms in this disease can be challenging or even overlooked, and diagnostic and treatment delays can lead to complications or sequelae, including infertility.

MDR-TB of the genitourinary tract is rare and difficult to diagnose. Timely diagnosis with the correct therapy, guided by a sensitivity test, is essential to achieve better outcomes and avoid future sequelae (e.g., kidney failure, infertility). This article describes the unusual case of a patient with MDR-TB epididymitis, which was successfully treated using a longer standardized regime.

## CASE REPORT

A 38-year-old male was evaluated at an outpatient clinic. He reported an 18-month history of increasingly painful testicular lumps associated with inflammation, which evolved to cutaneous fistula and secretion discharge. He did not report having other genitourinary and constitutional symptoms, such as fever and weight loss.

The patients’ diagnosis was made by isolating acid-fast bacilli (AFB) in urine samples through a microscopic examination. The urine samples were collected in a sterile wide-mouthed container for five consecutive days. The pooled urine specimens was centrifuged at 3000xg for 20 min. The resulting pellet was decontaminated with 4% NaOH. After being incubated for 15 min, the suspension was neutralized with phosphate-buffered saline (PBS; pH:6.8) and centrifuged again at 10,000 rpm for 20 min. The pellets of decontaminated urine were resuspended in PBS; smears were made for Ziehl-Neelsen staining. The GeneXpert/RIF (Xpert) assay detected Mtb with RIF resistance indeterminate and the culture tested positive for *Mycobacterium tuberculosis*. The patient underwent standard TB treatment with a four-drug regimen of isoniazid, rifampicin, ethambutol, and pyrazinamide (RHZE). After being treated for one week, the patient was hospitalized with acute liver injury and kidney failure, and had to undergo hemodialysis. The TB treatment was suspended and replaced by a regimen with levofloxacin and linezolid until liver and kidney functions recovered. On the 17^th^ day, the patient was discharged to continue follow-up and treatment in a TB reference center.

The patient had systemic lupus erythematosus and had been asymptomatic for the previous two years, without medication. Reports showed that he had been diagnosed with pleural TB 20 years before, achieving symptom remission after undergoing an RHZE treatment, even though he did not take his medication regularly. He was a former smoker (20 pack-years) and had quit two years before beginning the last treatment. He reported not having issues with drug abuse and not using any other substances. He was never imprisoned or homeless. He also reported having no family history of TB or other diseases and stated not performing risky or extramarital sexual activity in recent years.

During the first evaluation, the patient was afebrile, had a medium blood pressure of 83 mmHg, a pulse rate of 91 beats/min, and an oxygen saturation of 97% in room air. Lung sounds could be listened clearly during auscultation, the abdomen was innocent, and there was no peripheral lymphadenopathy. During genitalia examination, a lump was detected in the lower pole of the left testicle, underneath a thickened cutaneous area with phlogistic signs.

Laboratory tests presented the following results: hemoglobin = 11.5 g/Dl; white blood cell count = 4,700/mm^3^; platelet count = 259,000/mm^3^; creatinine level = 0.76 mg/dL; sodium concentration = 142 mg/dL; potassium concentration = 4.2 mg/dL; aspartate aminotransferase = 13 U/L; alanine aminotransferase = 19 U/L; uric acid 5.2 mg/dL; glucose level 84 mg/dL; and glycosylated hemoglobin 4.3%. The serologic test for HIV came back negative.

**Figure 1:**
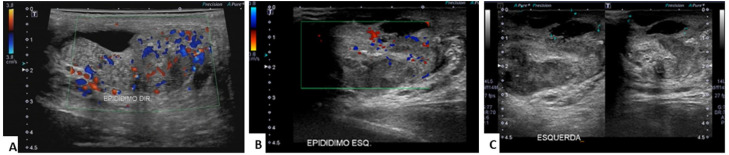
A) Ultrasound image showing a diffuse increase in the dimensions of the right epididymis and diffuse altered echotexture, with an increase in vascularization according to color Doppler mapping, compatible with epididymitis; B) Ultrasound image showing a diffuse increase in the dimensions of the left epididymis and diffuse altered echotexture, with an increase in vascularization based on color Doppler mapping, compatible with epididymitis; C) Ultrasound image showing signs of diffuse and heterogeneous increase in the dimensions of the left epididymis, exhibiting a small collection of liquid contiguous to the tail of the epididymis, situated on the left scrotal wall, which is thick and heterogeneous.

A testicular ultrasound revealed a thickened right epididymal (Figure 1A); a small foci of calcification in the left testicle; a heterogeneous expansive mass in the lower portion of the left testicle, associated with epididymis thickening and measuring around 7 cm on the longest axis (Figure 1B); and a septate liquid component (Figure 1C). The results of the chest computed tomography (CT) were normal. Abdominal CT scan showed interaortocaval and common iliac calcified lymph nodes without hepatosplenomegaly.

Given that the patient had previously had severe hepatotoxicity, professionals prescribed an alternative regimen containing linezolid, levofloxacin, and ethambutol for him. In the meantime, symptoms got worse: the patient started to experience fever, increasingly painful lumps, and fistula discharge, requiring surgical drainage. An anatomopathological analysis revealed a chronic granulomatous inflammatory process. An examination of the testicular fistula discharge showed positive bacilloscopy, a new rapid molecular test result was also positive for it, with low detection, and identified rifampicin resistance, the culture tested positive for *Mycobacterium tuberculosis*, and the Line Probe Assay (LPA) test found rifampicin and isoniazid resistance. Genital specimens were mixed with a 4 mL GeneXpert (Cepheid, Sunnyvale, CA) sample reagent, added to a GeneXpert cartridge and loaded onto the instrument. The results of this GeneXpert test were automated within approximately 2 h.

After professionals obtained these results, the patient began a directly observed 18-month therapy for multidrug-resistant TB (MDR-TB) with levofloxacin, terizidone, and ethambutol, combined with amikacin for the first eight months. This combination was the standardized regimen available for MDR-TB at the time. After the patient had undergone this treatment for one to four weeks, his clinical symptoms, drug tolerance, and kidney functions were evaluated, and he underwent liver enzyme monitoring. At the end of the treatment, patient’s laboratory test results were the following: creatinine level = 1.0 mg/dL, aspartate aminotransferase = 15 U/L, and alanine aminotransferase = 8 U/L. Then, patient went on to present reduced testicular mass and to achieve fistula resolution, but with persistent oligospermia.

## DISCUSSION

MDR-TB is a growing problem in countries with a high prevalence of TB. According to the World Health Organization, an overall increase in TB cases occurred due to the COVID-19 pandemic and its impact on TB detection. Recent data indicate that the MDR-TB rate is around 3.6% in newly diagnosed cases (primary resistance) and 18% in previously treated tuberculosis cases (acquired resistance)^1^. In Brazil, 1.5% of MDR- TB cases represent new TB cases, while 8% of MDR-TB cases are occur during TB retreatment^2^. According to the latest report, the number of MDR-TB cases in the Sao Paulo city has increased in the last years: 124 cases were recorded in 2022, while 89 were reported in 2019. Of all TB cases with resistance, 22.6% were MDR-TB^3^. Sputum AFB smear positivity, lung cavity, previously diagnosed or treated TB, HIV infection, diabetes mellitus, and smoking habits are considered risk factors for MDR-TB^4,5^. Molecular tests for detecting MTB DNA and mutations for resistance should be taken by all patients before initiating treatment, while waiting for a drug susceptibility test following culture, which requires up to six weeks of incubation^1^. Patients who receive TB treatment with poor adherence, inadequate dosing/intervals, or loss of follow-up, along with those who fail the treatment or relapse, are at increased risk of MDR-TB and should undergo a drug resistance evaluation^1^.

It has been estimated that 8 to 24% of TB cases worldwide are extrapulmonary (EPTB), i.e., they account for an average of 15% of total TB cases notified to WHO^1^. Extrapulmonary TB cases more frequently involve lymph nodes (40%), the abdomen (23%), and pleura (13%). The next most common instances of extrapulmonary TB are those affecting the genitourinary tract, the skeletal system, the central nervous system, tuberculous abscesses, breast TB, and laryngeal TB^6,7^. It is hard to point out the precise prevalence of Urogenital TB, due to its underreporting. However, professionals who estimated the prevalence of UG-TB did find that it varies broadly depending on the geographical region, ranging from 2 to10% in Western Europe and the USA, and from 15 to 20% in Africa, Asia, Eastern Europe, and the Russian Federation^8^. Patients with EPTB are usually older or immunocompromised, mainly presenting HIV, diabetes, corticosteroid or other immunosuppressant drug usage, chronic kidney failure, transplant recipients, and neoplasia^8^, ^9^. EPTB occurs when MTB bacilli from the lung are disseminated through the lymphatic and hematogenous systems, affecting one or multiple organs and producing various symptoms. EPTB is usually paucibacillary, and its infection sites might be challenging to access, which makes it difficult to obtain specimens for diagnosis^6^, ^7^. Thus, most patients receive anti-TB treatment without definitive microbiological results.

Scrotal TB is secondary to the hematogenous dissemination of MTB bacilli and affects the testes, epididymis, and vas deferens. Around 50% of patients with this disease initially have isolated epididymitis, which develops into testicular TB if left untreated. Epididymal TB damages the ejaculatory ducts, causing infertility. Compromised seminal vesicles lead to calculi or abscesses. The diagnosis of this condition requires high clinical suspicion, a CT scan of the affected organ or tissue, a chest CT evaluating concomitant pulmonary TB, and local specimen collection via fine needle aspiration or epididymal biopsy for microbiological and molecular identification^8^.

A study analyzing patients with testicular TB found that 25% of them also presented active pulmonary disease and that their systemic symptoms were usually related to other EPTB sites, such as the lungs, rather than to testicular TB itself^8^. An analysis of epididymal TB cases in 47 patients showed that most of them experienced testicular swelling and pain (44%), as well as epididymal enlargement (25%), that most did not present systemic symptoms (84%), and that only 17% of them had pulmonary imaging suggestive of TB^10^. In our case, the patient presented local symptoms but did not experience fever, weight loss, night sweats, or respiratory symptoms.

The average time necessary to diagnose these patients was 142 days, from the onset of. This delay contributes to disease progression and tissue damage, which results in kidney failure and infertility. Oligospermia occurs due to granulomatous destruction and obstruction in the epididymis or vas deferens^8^. Signs of TB infection include scrotal fistulae and a thin odorless pus discharge^8^, which were both present in the reported case. Misdiagnosis also leads professionals to postpone treatment and to perform several surgeries. A previous case report of disseminated TB presented as chronic epididymitis was conducted under the hypothesis of malignancy. Although TB was one of the differential diagnoses, an orchiectomy was still performed^11^. Our patient was diagnosed around 12 months after the onset of symptoms and continued to present oligospermia after undergoing adequate treatment.

## CONCLUSION

Testicular MDR-TB is a challenge due to the difficulty in diagnosing EPTB. The patient described in this case report had a positive bacilloscopy exam, a negative culture test, and an undetermined molecular test. However, his history of previous tuberculosis without adequate treatment adherence or follow-up was crucial in alerting clinicians to the possibility of MDR-TB. This case emphasizes the importance of using imaging and biopsies to characterize the lesion and proceed to microbiological identification, followed by drug resistance tests to prescribe the proper treatment. It also reinforces the fact that delayed diagnosis results in disease evolution and tissue destruction, causing more dysfunction and sequelae.
